# Ormosia excelsa *Benthseed asepsis for the initiation of an *in vitro

**DOI:** 10.1186/1753-6561-8-S4-P133

**Published:** 2014-10-01

**Authors:** Daniel Silva, Flavio Bruno, Angela Imakawa, Nádia Rapôso, Paulo Sampaio

**Affiliations:** 1 College of Health Sciences, University of Amazonas - Av. Carvalho Leal 1777, Cachoeirinha, 69065-001, Manaus/AM - Brazil; 2 Department of Tropical Horticulture, National Institute of Research in the Amazon - AV. André Araujo 2936, Petrópolis, 69011970, Manaus/AM - Brazil; 3 College of Technology, University of Amazonas - Av. Darcy Vargas 1200, Parque 10, 69065-020 Manaus/AM - Brazil

## 

*Ormosia excels *Benth. is an Amazon medicinal plant popularly known as Tento amarelo which belongs to the Fabaceae family and is used in the treatment and prevention of diseases, once its seed extract is being tested to fight tooth decays[1]. It is also used in the timber industry to make cudgels, banks and canoes. Moreover, the seeds are often used in popular craftsworks [2-4]. The aim of this study was to develop an aseptic protocol to determine the best type and concentration of bactericidal and fungicidal agents for disinfection of seeds. The study was conducted at the College of Technology/University of Amazonas Plant Tissue Culture Lab, Manaus/AM where 90 Tento amarelo seeds were obtained from the Native Seed Amazon Center. They were washed with neutral ODD^® ^detergent and rinsed with running water for a minute. Then, they were immersed in 0.2% (w/v) Derosal solution for an hour under 100 rpm constant orbital stirring, followed by a bath in a 70% ethanol solution for 1 minute and finally immersed in a hypochlorite sodium solution at 0.25%, 0.50% and 1.0% (w/v) concentrations respectively, for 30 minutes under the same stirring. Subsequently, the explants were washed three times with sterile distilled water and inoculated on MS sterile basal culture medium. After 30 days, the explants were evaluated for the presence or absence of fungi and bacteria, as well as their survival rate. The experimental design was completely randomized and statistical analysis used simple percentages. There was no statistically significant difference between treatments of hypochlorite sodium at 0.25% and 1.0% (w/v) concentrations, respectively since both provided the same level of alive and axenic explants (100%). It was observed that even with long period of exposure of the seeds to hypochlorite sodium and Derosal, these antimicrobial agents have been effective, since they are not toxic to the seeds of this kind. Even when exposed to high concentrations of these antimicrobial agents, the seeds remained alive and vigorous after germination. Treatments that present high and efficient decontamination effects are indicated for Tento amarelo seed asepsis as this species has a good performance for *in vitro *germination. We can come to the conclusion that it is possible to start an *Ormosia excelsa *Benth *in vitro *culture, from this asepsis.
